# Molecular epidemiology of *Theileria parva* in cattle, factors associated with the positivity in northern Malawi, and efficacy of traditional medicine

**DOI:** 10.3389/fmicb.2026.1826781

**Published:** 2026-08-28

**Authors:** Gift Kingsley Kayira, Takahiro Ishizaki, Shingo Asakura, Ryota Matsuyama, Makoto Ukita, Kyoko Hayashida, Haruyuki Hirata, Kohei Makita

**Affiliations:** 1 WOAH Collaborating Centre for Food Safety, Rakuno Gakuen University, Ebetsu, Japan; 2 Department of Animal Health and Livestock Development, Ministry of Agriculture, Lilongwe, Malawi; 3 International Institute for Zoonosis Control, Hokkaido University, Sapporo, Japan

**Keywords:** cattle, East Coast fever, ethnoveterinary, Malawi, *Synadenium*, *Theileria parva*, traditional medicine

## Abstract

**Introduction:**

East Coast fever (ECF), caused by *Theileria parva*, remains endemic in Malawi. ECF vaccines and chemical treatments are regarded as expensive by small-holder farmers. The aims of this study were to clarify the epidemiology of ECF and to evaluate the efficacy of ethnoveterinary medicine, *Synadenium cupulare* on *T. parva* infection.

**Methods:**

A cross-sectional study was conducted in three districts of northern Malawi (Chitipa, Karonga, and Mzimba). A structured questionnaire on feeding style, treatment of ECF, and tick control practices was administered to 79 farmers, and blood samples and animal information were collected from 550 cattle. PCR using p104 gene-specific assay was used to screen for *T. parva*, and positive samples were subjected to semi-nested PCR using Tp1 and Tp2 primers to characterize their genetic traits. Descriptive statistics were conducted. Global spatial clustering was tested using Moran’s *I*. Univariable and multivariable statistical analyses were performed on farm- and animal-level factors associated with *T. parva* positivity.

**Results:**

The overall farm- and animal-level prevalence was 78.5% (62/79 farms, 95% CI: 67.8–86.9%) and 28.4% (156/550 animals, 95% CI: 24.6–32.3%), respectively. Phylogenetic trees suggested 4 variants of Tp1 and 6 variants of Tp2 circulations among cattle populations in Malawi. Moran’s *I* showed clustering of *T. parva* negative farms only in Karonga District (*p* < 0.001). Farm-level *T. parva* prevalence was significantly higher in the farms conducting extensive grazing (81.9%) than those not (42.9%, *p* = 0.035). At the animal level, the final multivariable generalized linear mixed-effects model included age and treatment types. For age, the odds of *T. parva* positivity were significantly lower in adults (odds ratio [OR] = 0.47, *p* = 0.003) compared with calves, but not for weaners (OR = 0.78, *p* = 0.316). Regarding treatment, the odds of *T. parva* positivity were significantly higher in untreated cattle with symptoms of ECF (OR = 2.38, *p* = 0.027) compared with asymptomatic cattle, but no significant increase was observed for those treated with oxytetracycline (OR = 0.99, *p* = 0.971) or *S. cupulare* (OR = 0.59, *p* = 0.133).

**Discussion:**

Future ECF control should integrate traditional ethnoveterinary medicine for calves.

## 1 Introduction

Among the major diseases affecting cattle in sub-Saharan Africa, tick-borne diseases (TBDs) are of particular concern, especially in regions where cattle are maintained under open grazing conditions (Rubaire-Akiiki et al., 2004). Among all known TBDs, East Coast fever (ECF) is one of the most devastating diseases affecting cattle in central, eastern, and southern Africa (Otim, 2000; Lawrence et al., 2004). Notably, ECF is estimated to cause the death of at least 1 million cattle annually across the continent (Olwoch et al., 2008), and therefore constitutes a serious threat to livestock production.

ECF is caused by the apicomplexan protozoan *Theileria parva* and is maintained in a multi-host transmission system. It is transmitted mainly by ixodid ticks, *Rhipicephalus appendiculatus*, and by *R. zambeziensis* in some areas (De Meneghi et al., 2016). The natural reservoir hosts of *T. parva* are African Cape buffalo (*Syncerus caffer*) (Moumouni et al., 2015) and waterbuck (*Kobus ellipsiprymnus*). However, under pastoral production systems, traditionally managed cattle constitute the principal reservoir of *T. parva* and play a key role in sustaining transmission (Perry and Young, 1995).

Clinical outcomes of ECF infection in cattle are heterogenous. Infected cattle may exhibit mild, moderate, or severe clinical symptoms such as loss of appetite, enlarged lymph nodes, fever, lacrimation, nasal discharge and respiratory distress. Those exhibiting mild or moderate symptoms are considered relatively resistant and often recover clinically, however, they can remain persistently infected and serve as a source of infection for ticks (Kariuki et al., 1995). In contrast, cattle developing severe clinical signs are considered susceptible and may progress to a highly fatal form of the disease. Mortality rates of 40–80% have been reported in Maasai pastoralist herds (Homewood et al., 1987).

Management of ECF involves both preventive and therapeutic approaches, often requiring a balance between effectiveness and cost. Chemical acaricides have long been applied through plunge-in dipping, hand spraying, and pour-on products (Minjauw and McLeod, 2003). However, acaricides are costly, cause environmental contamination, and prolonged, high-frequency use of the same compounds has led to the emergence of acaricide resistant ticks (Miyama et al., 2020). In Malawi, plunge-in dipping, which was previously conducted by government agencies to support smallholder cattle farmers, has not been practiced since the early 2000s. Immunization using the Muguga cocktail vaccine is also employed as a preventive measure against ECF (Radley et al., 1975; Toye et al., 2020). Although the Muguga cocktail vaccine is available to cattle farmers, its high cost ($10 USD per animal) limits widespread adoption (Lynen et al., 2012). Some of the major *T. parva* antigens, such as Tp1 and Tp2, are known to induce CD8 + T cell response in cattle, however, protective immunity is acquired only in a subset of immunized animals expressing specific major histocompatibility complex (MHC) class I alleles (MacHugh et al., 2009). Understanding the distribution and genetic diversity of major *T. parva* antigens is thus important for future vaccine design. With regard to ECF treatment, tetracycline is among the earliest compounds used over several decades (Perry and Young, 1995). However, its therapeutic efficacy is limited to the early stages of infection. In the late 1970s, naphthoquinone derivatives (parvaquone and buparvaquone) were identified as more effective treatments. Nevertheless, the high cost of these drugs discourages their use under field conditions (Gachohi et al., 2012).

The livestock industry plays a critical role in Malawi’s economy, contributing approximately 11% of the national gross domestic product (GDP) and approximately 36% of agricultural GDP (DAHLD, 2021). Consequently, ECF represents a major challenge to livestock production in the country. Although ECF has traditionally been considered non-endemic in the southern Malawi, where approximately 71% of the national dairy cattle population is raised (DAHLD, 2021), recent geographical spread of *T. parva* infestation has been reported (Chikufenji et al., 2024). In contrast, ECF is endemic in the central and northern Malawi, where case-fatality rate in calves of up to 46% has been reported (Solden, 2009). Exotic cattle breeds are highly susceptible to ECF across all age groups unless rigid vector control measures are implemented (Bishop et al., 1992), and high morbidity and mortality have been reported among Friesian dairy cattle introduced to smallholder farmers in northern Malawi (Musisi et al., 1996; Kumwenda and Mingu, 2005).

**Figure 1:**
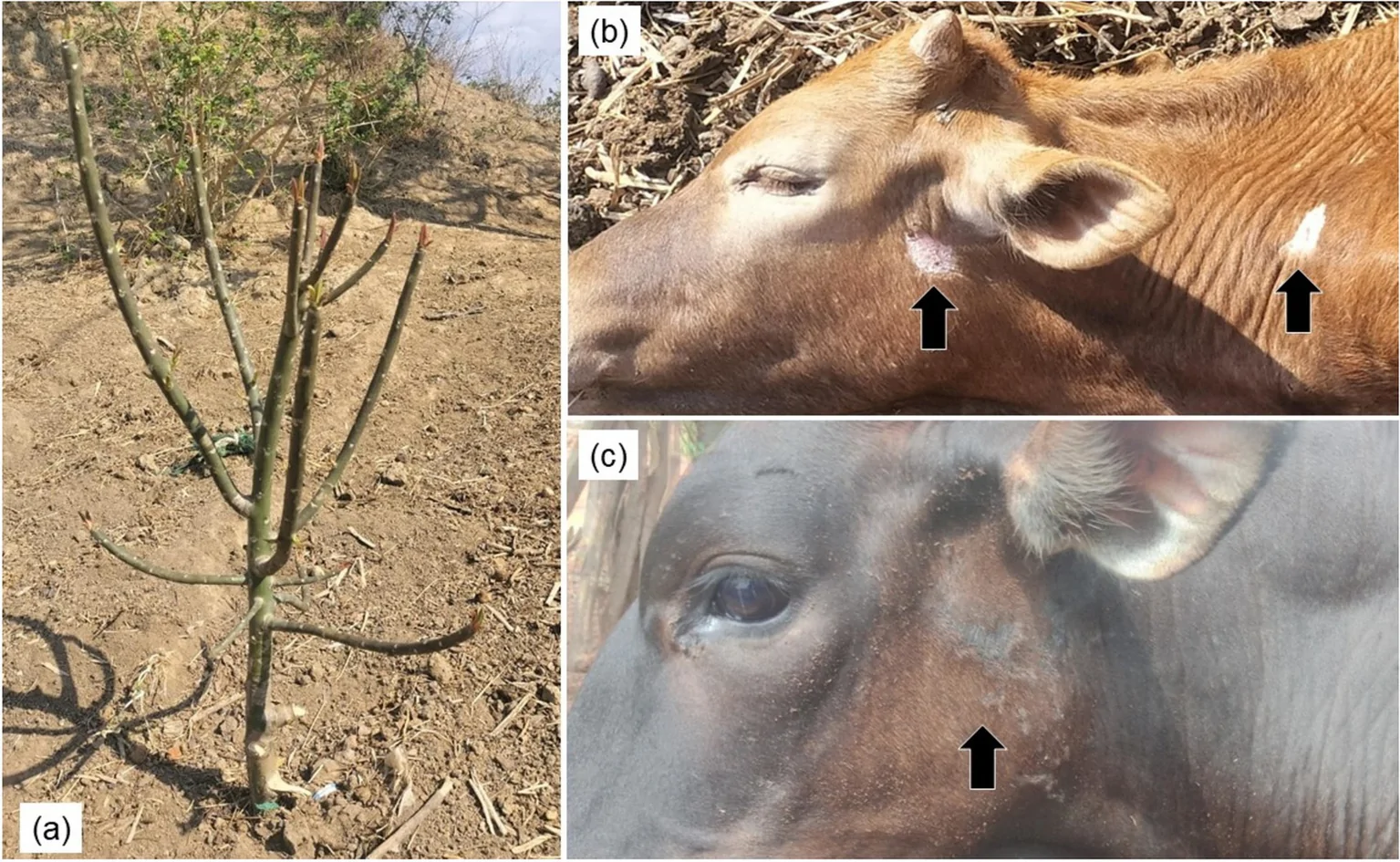
Photographs of *Synadenium cupulare* **(a)** and scars of *S. cupulare* application on the skin of calves **(b, c)**.

Due to the above-mentioned high costs of chemical treatment or vaccine (Young et al., 1990), alternative approaches including ethnoveterinary remedies have been adopted among smallholder farmers with limited access to formal veterinary services in Malawi. For example, *Synadenium cupulare*, a member of the *Euphorbiaceae* family plant (Figure 1a), is widely used during the early stage of ECF infection by resource-limited farmers in several East African countries, including Kenya and Uganda (Gakuubi and Wanzala, 2012; Ndizihiwe et al., 2019). In Malawi, latex from *S. cupulare*, is traditionally applied to cattle following skin incisions close to lymph nodes (Figures 1b,c), though its efficacy against ECF remains poorly defined (Kayira, 2025). In this study, we characterized the molecular epidemiology of ECF and investigated factors associated with ECF occurrence and evaluated the effect of the ethnoveterinary use of *S. cupulare* against ECF in northern Malawi.

## 2 Materials and methods

### 2.1 Study area

**Figure 2:**
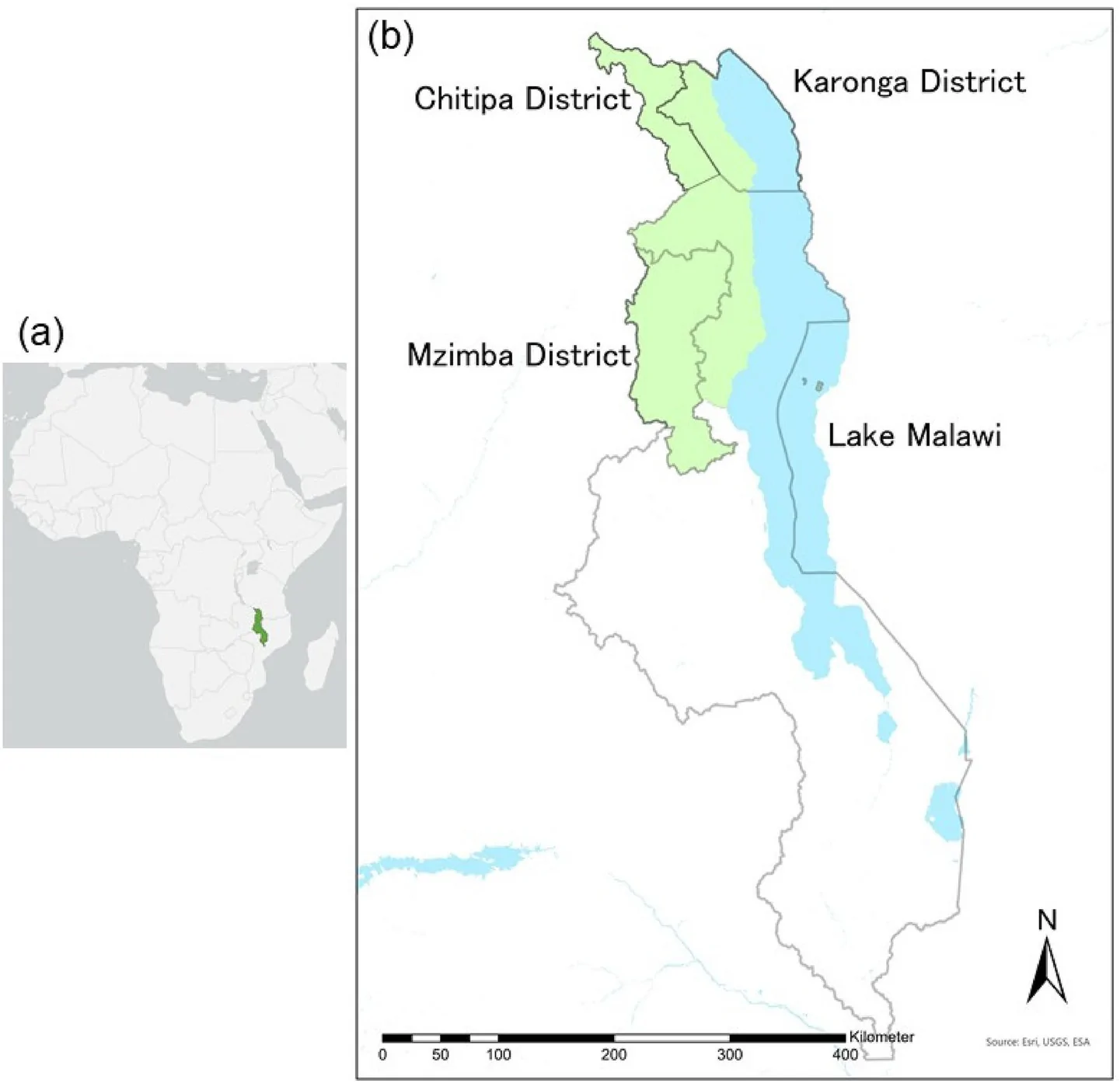
Maps of Africa with Malawi indicated **(a)** and the study areas with Northern Region indicated with green color **(b)**.

The study was conducted in the Chitipa (Lufita and Misuku Extension Planning Areas: EPAs), Karonga (Mpata and Kaporo South EPAs), and Mzimba (Emsizin, Njuyu, Mpherembe, Mbalachanda, and Zombwe EPAs) districts of the northern region of Malawi (Figure 2). Malawi, located in the southern part of Africa, is a landlocked, agriculture-based country covering 118,484 km^2^. The northern part of Malawi is characterized by a savanna tropical climate with a hot, dry portion of summer (September–November) a hot, wet portion of summer (December–April), and dry winter (May–August). The mean annual rainfall of Malawi ranges from 725 mm to 2,500 mm (MNRCC, 2010), and monthly mean temperatures varies between 17 and 27 °C (World Bank Group, 2026).

### 2.2 Study design

The study employed a cross-sectional design. The sample size was calculated based on comparing two proportions: the prevalence of *T. parva* in herds treating animals using *S. cupulare* and that in herds not treating animals (Equation 1) (Dohoo et al., 2004):

$$n=\frac{{\left(Z_{\alpha }\sqrt{2\mathrm{pq}}-Z_{\beta }\sqrt{p_{1}q_{1}+p_{2}q_{2}}\right)}^{2}}{{\left(p_{1}-p_{2}\right)}^{2}}$$ (1)

where *n* = sample size in a group, $Z_{\alpha }$ = multiplier for type I error (deviate in a standard normal distribution at 97.5 percentile: 1.96, 5% level of significance for two-sided tests), $\;Z_{\beta }$ = multiplier for type II error at the power of 80%, $p_{1}$ = an a-priori prevalence of 35% in non-treated animals, and $p_{2}$ = an a-priori prevalence of 30% in *S. cupulare* treated, given the reported *T. parva* prevalence in Malawi at 33% (Chatanga et al., 2022) (note: two-sided $Z_{\alpha }$ was selected as the efficacy of *S. cupulare* has not been proved yet in experiments). Therefore, the sample size was calculated to detect 5% difference in *T. parva* prevalence between two groups. $p=(p_{1}+p_{2})/2$ , and $q_{1}$ , $q_{2}$ , and *q* are the complements of $p_{1}$ , $p_{2}$ , and *p*, respectively. The resulting minimum required sample size in one group was 220, and for comparison, 440 animals were needed.

Next, assuming that 20% of smallholder farmers use chemical treatment for ECF, to allow assessing the ethnoveterinary effect of *S. cupulare* among farmers not using chemical drugs, the calculated sample size 440 was divided by 0.8 (a complement of 20% using chemical treatment), and the sample size was adjusted as 550.

Considering farm-level analysis, the number of animals to be sampled in a herd, $n_{\det}$ , was calculated based on the detection of *T. parva* positive animals using Equation 2 (Dohoo et al., 2004):

$$n_{\det}=(1-\alpha^{\frac{1}{D}})(N-\frac{D-1}{2})$$ (2)

where α = 1—confidence level, 0.05, D = estimated minimum number of diseased animals in an assumed herd of 15 animals which is representative of the study area (35% of 15 animals = 5.25 ≈ 5), and N = population size = 15 animals in a herd. $n_{\det}$ was calculated as 6 (5.86), and to further ensure assessing within-herd epidemiology as well, 7 animals per herd were planned to be sampled. Finally, the number of herds to be sampled was calculated as 79, dividing 550 by 7 animal to be sampled per herd.

A multistage random sampling at EPA and section levels was used to select the herds. Smallholder cattle farmer registration data were used for the selection of sampling farms.

### 2.3 Field survey

#### 2.3.1 Interviews using a questionnaire

Interviews using a structured questionnaire were conducted in dry seasons between February 2024 and December 2024 across 79 cattle farms in 9 EPAs. The questionnaire written in the local language (*Chitumbuka*) was administered to cattle farmers through face-to-face interviews. The questionnaire was designed to collect information on herd size, symptom-based incidence of ECF, prevention and treatment strategy employed, tick control practices including ethnoveterinary treatment, and access to veterinary services. Livestock farming system (extensive grazing or semi-intensive, which cut and carry grass to the farms) and presence or absence of ticks on cattle were observed and recorded as herd-level data.

#### 2.3.2 Collection of blood samples

Blood sampling was conducted during the same time as the interviews using a questionnaire. Cattle were purposively selected so that the age structure represents the herd. Animals that were in the late stages of pregnancy (second and third trimesters) were excluded from sampling to avoid inducing stress in the animals. Blood was collected from the animals via the jugular vein.

The collected animal-level data included age, sex, breed, health status at time of sampling (sick or healthy), body condition score (BCS), and any treatment given in the previous 12 months. For situations in which a farmer had no record of the age of an animal, dentition was evaluated by Assistant Veterinary Officers (AVOs) to estimate the animal’s age (Torell et al., 2003). BCS was recorded over the range 1–5, with 1 = very poor and 5 = Obese (Ferguson et al., 1994). All selected cattle owners consented to participate in the questionnaire and allowed sampling of their animals.

The collected blood samples were temporally kept on ice in the field and transported to Mzuzu Veterinary Laboratory, where they were stored at −20 °C until DNA extraction. Sampling was conducted in two phases. During the first phase (February–March 2024, *n* = 202), each collected blood sample was spotted onto a Whatman Fast Technology for Analysis of nucleic acids (FTA)™ card (Merck KGAa, Darmstadt, Germany) in accordance with the manufacturer’s instructions and subsequently transported to Rakuno Gakuen University for DNA extraction and PCR analysis. During the second phase (November to December 2024, *n* = 348), blood samples were collected into EDTA tubes, DNA was extracted at the Mzuzu Veterinary Laboratory, and FTA cards were not used.

### 2.4 Molecular analysis

#### 2.4.1 DNA extraction

For the first 202 blood samples applied to Whiteman FTA cards, genomic DNA was extracted using the DNeasy blood and tissue kit (QIAGEN, Hilden, Germany), following the manufacturer’s protocol. For the remaining 348 samples, DNA was extracted from 200 μL of whole blood collected in the EDTA tubes using the same kit. Both FTA card application and EDTA pre-treatment were reported to have no influence on the sensitivity of detection of protozoan parasites (Okino et al., 2018). All extracted DNA samples were stored at −30 °C before use.

#### 2.4.2 p104 PCR for the detection of *T. parva*

The presence of *T. parva* was determined using a nested PCR assay targeting the *T. parva* 104-kDa antigen gene (p104), as described previously (Odongo et al., 2010; Skilton et al., 2002). The primers used during the PCR are presented in Table 1. First PCR reactions were performed in a total volume of 20 μL containing 10 μL of 2 × Gflex PCR Buffer (Mg^2+^, dNTP plus), 0.4 μL of Tks Gflex DNA polymerase (TaKaRa Bio Inc., Shiga, Japan), 200 nM of each forward and reverse primer, 7.6 μL molecular-grade water, and 1 μL of DNA template. The thermal cycling conditions consisted of an initial denaturation at 94 °C for 1 min, followed by 35 cycles of denaturation at 98 °C for 10 s, annealing at 60 °C for 15 s, extension at 68 °C for 1.5 min, and final extension at 68 °C for 5 min. Second PCR reactions were conducted using the same reaction volumes and reagent concentrations as those used for the first PCR, except for the primer set and the template. The cycling conditions for the second PCR included initial denaturation at 94 °C for 1 min, followed by 30 cycles of denaturation at 98 °C for 10 s, annealing at 55 °C for 15 s, extension at 68 °C for 1 min, with a final extension at 68 °C for 5 min. PCR products were separated by electrophoresis on 1% agarose gel, pre-stained with Gel-Red (Biotium, Hayward, CA, USA), and visualized under blue light transilluminator. To minimize false-positive results, the PCR detection threshold was determined with ImageJ software, version 1.53 t (Wayne Rasband, National Institutes of Health, Bethesda, MD, USA) (Schneider et al., 2012).

**Table 1:** List of primers for detection of *Theileria parva* in this study.

Primer name	Primer sequence (5′–3′)	Target gene/(PCR type)	Product size (bp)	Anneal. T.(C)	References
IL 3231	ATTTAAGGAACCTGACGTGACTGC	*T. parva* p104 gene PCR	496	55	Kariuki et al. (1995)
IL 755	TAAGATGCCGACTATTAATGACAC				
IL4234	GGCCAAGGTCTCCTTCAGAATACG	*T. parva* p104 gene nested PCR	277	60	Perry and Young (1995)
IL3232	TGGGTGTGTTTCCTCGTCATCTGC				
Tp1 F outer	ATGGCCACTTCAATTGCATTTGCC	Tp1 gene PCR	432	50	Radley et al. (1975)
Tp1 R outer	TAAATGAAATATTTATGAGCTTC				
Tp2 F outer	ATGAAATTGGCCGCCAGATTA	Tp2 gene PCR	525	50	Radley et al. (1975)
Tp2 R outer	CTATGAAGTGCCGGAGGCTTC				
Tp1 Inner F1	CCGCKGATCCTGGATTCT	Tp1semi-nested PCR	419	60	Radley et al. (1975)
Tp1 R outer	TAAATGAAATATTTATGAGCTTC				
Tp1 Inner F2	CATTTGCCGCKGATCCTG	Tp1 gene alternative semi-nested PCR	413	55	
Tp1 R outer	TAAATGAAATATTTATGAGCTTC				
Tp2 Inner F	CCGCCAGATTAATTAGCCTTT	Tp2 gene semi-nested PCR	511	58	
Tp2 R outer	CTATGAAGTGCCGGAGGCTTC				

F, forward; R, reverse; PCR, polymerase chain reaction.

#### 2.4.3 Tp1 and Tp2 PCR for characterization of *T. parva*

Samples positive for *T. parva* p104 were subjected to semi-nested PCRs targeting the Tp1 and Tp2 gene, with gene-specific primers (Table 1). In brief, the first and second PCR reactions were performed using the same reaction volume and conditions as those used for the p104-targeting PCR described above. The thermal cycling conditions for the first PCR of Tp1 and Tp2 were an initial denaturation at 94 °C for 1 min, followed by 35 cycles of denaturation at 98 °C for 10 s, annealing at 50 °C for 15 s, extension at 68 °C for 30 s, and a final extension at 68 °C for 5 min. The thermal cycling conditions for the second PCR of Tp1 are the same as described above, except the annealing temperature is 55 °C. The PCR conditions for Tp2 are the same as described above, except the annealing temperature is 57 °C (Chatanga et al., 2020). The resulting amplicons were purified using NucleoSpin Gel columns and a PCR Clean-Up kit (Macherey-nagel, Düren, Germany). Sequencing was carried out in both directions using a BigDye™ Terminator v3.1 Cycle Sequencing kit (Applied Biosystems, Foster, California, USA) and ABI genetic analyzer 3500xl (Applied Biosystems).

#### 2.4.4 Sequence and phylogenetic analysis

Raw sequencing data were edited using the ‘A plasmid Editor’ (ApE) software (Wayne and Jorgensen, 2022) by trimming primer annealing sites, and consensus sequences were generated for amino acids alignment and phylogenetic analysis. The nucleotide sequences obtained in this study were deposited in the Genbank under the following accession numbers: PX418011-PX418014 for Tp1 and PX418015 – PX418020 for Tp2. Nucleotide alleles and translated amino acid variants were named in accordance with previously established nomenclature, which defines nucleotide alleles 1–49 and amino acid variants 1–34 for Tp1 and nucleotide alleles 1–63 and amino acid variants 1–59 for Tp2 (Pelle et al., 2011).

Single-nucleotide polymorphisms (SNPs) were identified in the Tp1 and Tp2 sequences obtained in this study. To assess the phylogenetic relationships between the sequences obtained here and those available in Genbank, maximum-likelihood phylogenetic trees were constructed using Molecular Evolutionary Genetic Analysis (MEGA 12) software with the Tamura two-parameter substitution model (Kumar et al., 2016).

### 2.5 Statistical analysis

Descriptive epidemiology analyses were conducted. The 95% confidence intervals of farm and animal-levels prevalence values were estimated using a one-sample proportions test with continuity correction. Spatial clustering of *T. parva* positivity and use of *S. cupulare* was tested using global Moran’s *I* in R package “ape” (Emmanuel and Bolker, 2025).

Univariable analysis was conducted for the farm-level risk factors for *T. parva* positivity using the Chi-square tests or Fisher’s exact tests. The factors associated with the within-farm *T. parva* positivity were analyzed using generalized linear models with binomial errors using the data of *T. parva* positive farms. Univariable analyses were conducted to examine the association between animal-level positivity for *T. parva* and categorical attributes such as districts, age group, sex, breed, and treatment using either the Chi-square test or Fisher’s exact test. BCS was compared between *T. parva* positive and negative animals using Wilcoxon rank sum test. To further understand associations between variables in relation to *T. parva* positivity, multiple comparisons were performed. Multi-variable risk factor analysis for *T. parva* positivity at animal level was conducted using generalized linear mixed-effects models with binomial errors, selecting farm identification as a random effect, employing backward stepwise model simplification. All statistical analyses were conducted using R statistics software, version 4.3.1 (R Core Team, 2024).

## 3 Results

### 3.1 Characteristics of the cattle herds studied

In total, 550 cattle were sampled on 79 farms. The farms were in Chitipita (129 cattle on 22 farms), Karonga (92 cattle in 13 farms), and Mzimba Districts (329 cattle in 44 farms). The mean herd size was 14.6 cattle (median = 13.0, interquartile range [IQR] range = 9.5–18.0, range = 4–35). The mean age of sampled cattle was 16.2 months (median = 10.0 months, IQR = 6.0–18.0 months, range = 3–120 months). Most farms were characterized by extensive grazing system (72/79 farms, 91.1%) and the remaining farms by semi-intensive (7 farms, 8.9%).

Tick control using acaricide hand spraying was practiced on 30.4% (24 farms) of the farms, and the frequency of spraying was fortnightly in 5 farms (20.8%), monthly in 9 farms (37.5%), and once in 2 months in 10 farms (41.7%). With regard to perceived vector-borne diseases, the most common was ECF (69.6%, 55/79 farms), followed by babesiosis (16.5%, 13/79) and trypanosomiasis (2.5%, 2/79). There were 9 farms without perception of vector-borne diseases. Farmers were able to state clinical signs of TBDs in *Chitumbuka*, the local language.

Regarding cattle breed, of the 72 extensive farms that conducted grazing, 71 had Malawi zebu cattle (98.6%), and one farm had Malawi zebu–Friesian cross breed cattle. Among the 7 semi-intensive farms, 4 had Malawi zebu–Friesian cross breeds (51.7%), 1 had pure Friesian cattle, and 2 had Bosmara cross breed cattle. At the animal level, Malawi zebu was the most dominant (92.0%, 506/550 animals), followed by Malawian zebu-Friesian cross (5.6%, 8 animals). Pure Friesians represented 1.5% (8/550 animals), and Bosmara–Malawi zebu crossbreeds 0.9% (5 animals).

### 3.2 Practice of treatment against East Coast fever

For the treatment of ECF, 44 farms (55.7%, 44/79 farms) used oxytetracycline (OTC), and 25 farms *S. cupulare* (31.6%, 25/79 farms). No farms reported applying both methods together, and 10 farms never treated cattle against ECF.

Drugs were primarily administered by AVOs and Animal Health Surveillance Assistants (AHSAs) on farms that used OTC, whereas Community Animal Health Workers (CAHWs) predominantly administered treatments on farms that relied on local remedies. AVOs and AHSAs are government employees, whereas CAHWs are private community-based animal health workers that have received formal training from government veterinary experts. There were no significant differences between the proportions of treatment options (no treatment, OTC, or *S. cupulare*) between extensive grazing and semi-intensive farms (Fisher’s exact test, *p* = 1, Table 2).

**Table 2:** A comparison of the treatment options against East Coast fever between extensive gazing and semi-intensive farms.

Treatment option	Number of extensive grazing farms (*n* = 72)	Percentage	Number of semi-intensive farms (*n* = 7)	Percentage
No treatment	9	12.5%	1	14.3%
Oxytetracycline	40	55.6%	4	57.1%
*Synadenium*	23	31.9%	2	28.6%

Regarding differences in treatment practice by cattle breed, all the pure Friesian cattle (*n* = 2) and Malawi zebu–Friesian crossbreed cattle (n = 12) showing symptoms were treated, whereas 16.9% (31/183) of Malawi zebu cattle exhibiting symptoms were not treated. Table 3 summarizes the relationships between feeding style, treatment options, and *T. parva* positivity at the animal level. The *T. parva* positivity rate did not significantly differ between extensive farms that conducted communal grazing and semi-intensive farms that used a cut and carry system for animals not exhibiting symptoms of ECF (28.3% vs. 23.7%, *χ*^2^ = 0.162, df = 1, *p* = 0.687), for cattle treated with OTC (27.1% vs. 33.3%, *p* = 0.735 by Fisher’s exact test) or *S. cupulare* (20.4% vs. 25.0%, *p* = 1 by Fisher’s exact test).

**Table 3:** Cross-tabulation of feeding management style and treatment option in *Theileria parva*-positive animals.

Feed management	No symptoms	Not treated	Oxytetracycline	*Synadenium*
Extensive grazing	28.3% (89/315)	51.6% (16/31)	27.1% (26/96)	20.4% (11/54)
Semi-intensive	23.7% (9/38)	0% (0/7)	33.3% (4/12)	25.0% (1/4)

### 3.3 *Theileria parva* prevalence and spatial distribution in cattle farms

**Figure 3:**
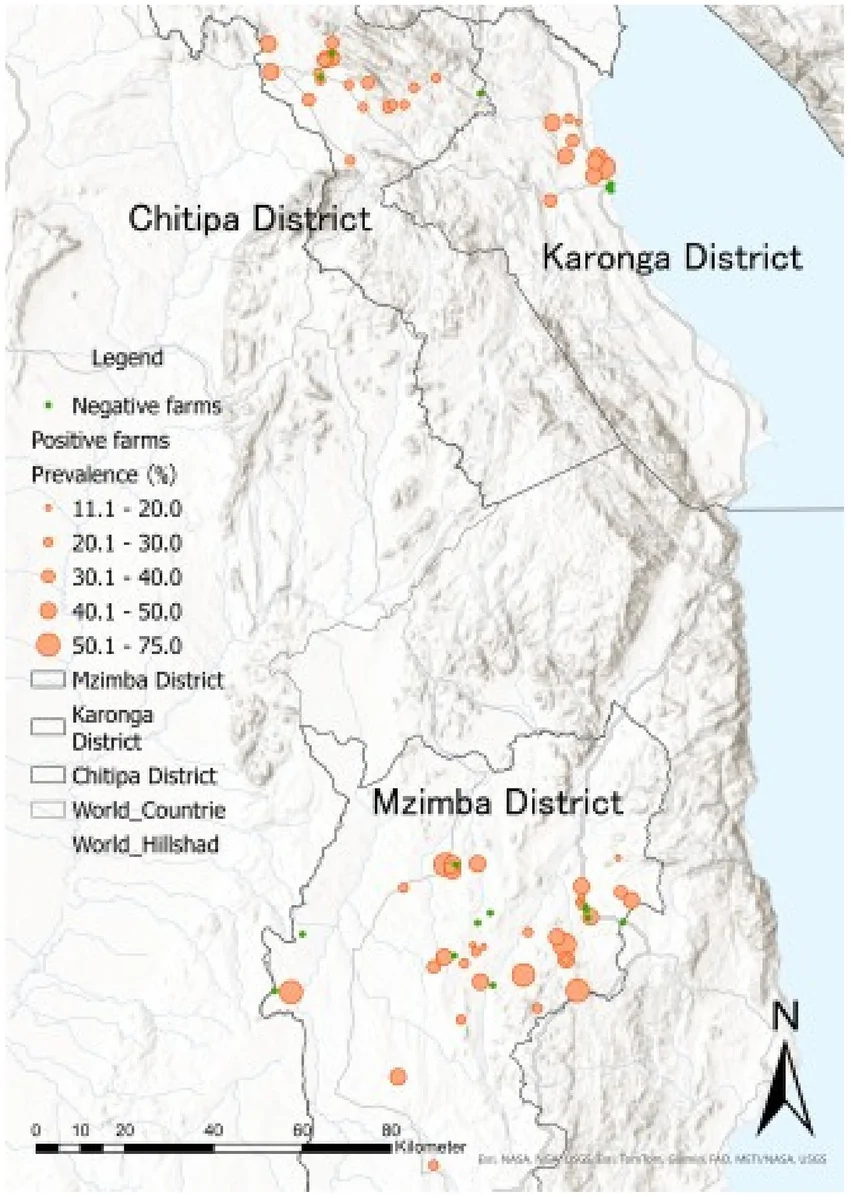
A map of the *Theileria parva* positive and negative farms with within-farm prevalence indicated as the size of symbol. The size of red circles shows the within-farm prevalence of *T. parva*.

The farm- and animal-level prevalence of *T. parva* were 78.5% (62/79 farms, 95% CI: 67.8–86.9%) and 28.4% (156/550 animals, 95% CI: 24.6–32.3%), respectively. The mean, median, range and IQR of within-farm prevalence values were 28.3, 28.5%, 0.0–75%, and 14.3–42.8%, respectively. Figure 3 shows the geographical distributions of *T. parva* positive and negative farms with the size of red circles indicating within-herd prevalence. Global Moran’s *I* indicated a spatial cluster of *T. parva* only in Karonga District (*p* < 0.001) but in Chitipa (*p* = 0.470) and Mzimba Districts (*p* = 0.961). In Karonga District, out of 13 farms, only 3 farms (2 extensive-grazing farms and a semi-intensive farm) were *T. parva* negative, and these farms located close each other (Figure 3). No global clustering was observed in the use of *S. cupulare* in all the Chitipa (*p* = 0.620), Karonga (*p* = 0.129), and Mzimba Districts (*p* = 0.125).

### 3.4 Sequence variation and phylogenetic relationships of *Theileria parva* Tp1 and Tp2

*T. parva–*positive samples were selected based on sampling area and subjected to semi-nested PCR using Tp1 and Tp2 primers. In total, 28 Tp1 and 37 Tp2 nucleotides were successfully amplified. SNPs were detected in both Tp1 and Tp2, indicating sequence variation at individual nucleotide positions; however, no nucleotide deletions resulting in amino acid substitutions within the cytotoxic T lymphocyte (CTL) epitope region of Tp1 were observed.

This study identified two and three novel nucleotide alleles for Tp1 and Tp2, respectively, leading to the detection of previously unreported antigen variants in the cattle population examined. Amino acid sequence alignment revealed both conserved and variable regions, and five antigen variants were identified and designated Var-16 (*n* = 11), −35 (*n* = 3), −37 (*n* = 8), −41 (*n* = 1), and −44 (*n* = 5) according to the nomenclature proposed by Pelle et al., 2011. Similarly, analysis of Tp2 amino acid sequence alignment revealed conserved and variable regions, and seven antigen variants were identified and designated Var-1 (*n* = 25), −3 (*n* = 3), −37 (*n* = 2), −61 (*n* = 2), −63 (*n* = 1), −67 (*n* = 1), and −68 (*n* = 3). Detailed descriptions of the epitope variants identified in this study are reported elsewhere (Kayira, 2025).

**Figure 4:**
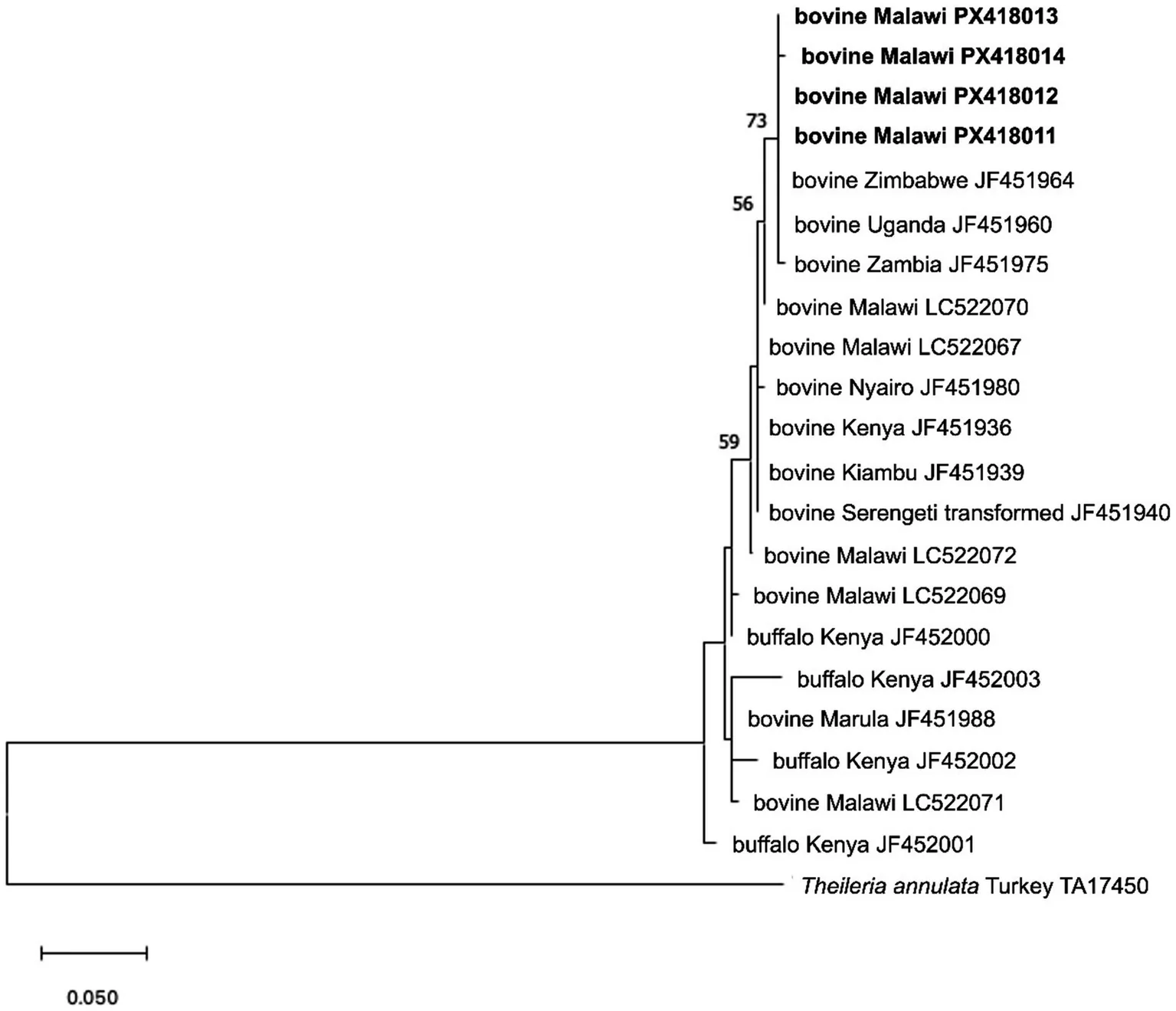
A phylogenetic tree for Tp1 gene sequences indicating relationships between the *Theileria parva* isolates in this study and previously reported. The isolates in this study formed a single cluster including the isolates from bovine in Zambia, Zimbabwe, and Uganda.

**Figure 5:**
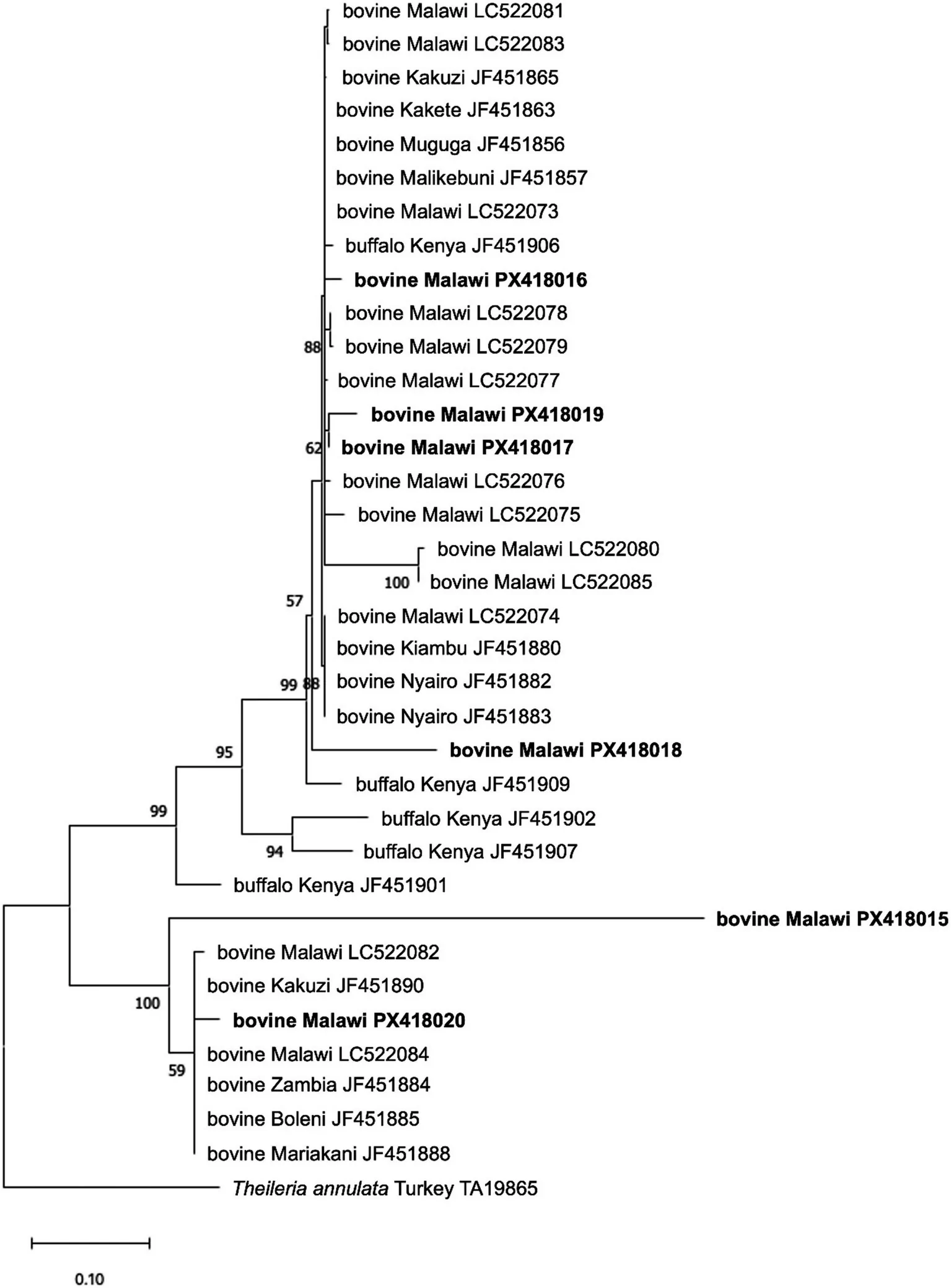
A phylogenetic tree for Tp2 gene sequences, indicating relationships among the cattle-derived *Theileria parva* isolates. The isolates formed two subclades (top: PX418016 – 418018, and bottom: PX418015 and 418020) with a diversity scattered across the clusters.

**Figure 6:**
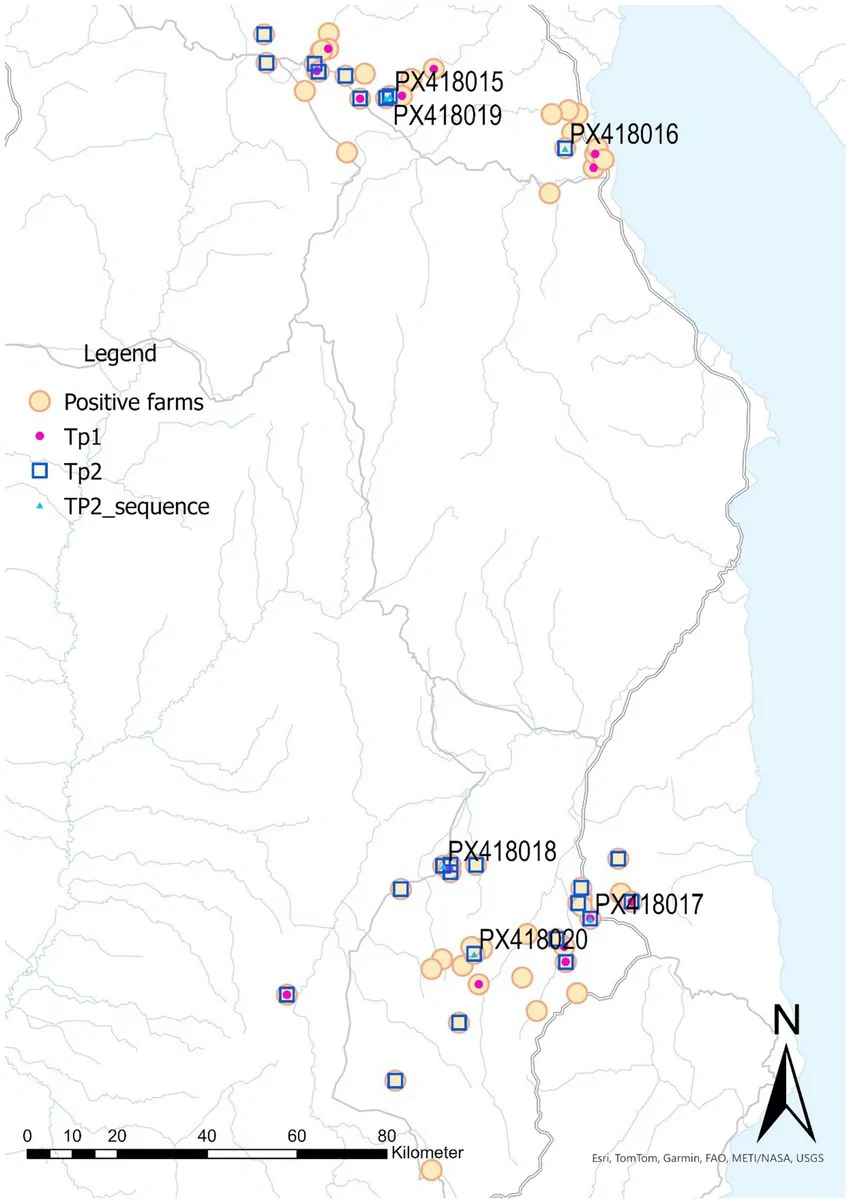
A map showing the geographical distributions of *Theileria parva* positive farms, with Tp1 and Tp2 detected, and Tp2 genome sequence was read. Yellow circle indicates *T. parva* positive farms. Out of these, pink dots and blue squares show the locations of the farms with cattle from which Tp1 and/or Tp2 were detected. Blue triangles and the accession numbers show the locations of farms where Tp2 genome sequence was read.

Phylogenetic analyses based on Tp1 and Tp2 gene sequences were subsequently performed to assess the genetic diversity between *T. parva* detected in this study and previously reported isolates. For the Tp1 gene, four sequences obtained in the present study (accession numbers PX418011-PX418014) clustered within the *T. parva* clade and grouped closely with reference sequence derived from African cattle isolates (Figure 4). These Tp1 sequences formed a well-supported monophyletic cluster, indicating that the detected parasites are genetically consisted with canonical *T. parva*. No distance sub-structuring or host-specific clustering was observed among the Tp1 sequences generated in this study. Similarly, phylogenetic analysis of the Tp2 gene demonstrated the six sequences obtained in this study (PX418015-PX418020) were clearly positioned within the *T. parva* lineage. These Tp2 sequences clustered with previously reported *T. parva* isolates from both cattle and buffalo and were clearly separated from other *Theileria* species. The Tp2 phylogenetic tree revealed two subclades (top: PX418016-PX418019, below: PX418015, PX418020) and minor sequence variation, scattered across the clusters, was observed among the Tp2 sequences, however, no clear geographical or host associated differentiation was detected (Figure 5). Notably, the Tp2 sequence PX418015 exhibited a longer branch length, suggesting higher genetic divergence and increased polymorphism relative to the other sequences. Overall, phylogenetic analysis of both Tp1 and Tp2 genes consistently confirmed the parasites detected in this study belong to *T. parva* demonstrating genetic homogeneity with previously characterized *T. parva* populations. The farms with cattle from which Tp1 and/or Tp2 were isolated were geographically distributed in all three districts. The association between genetic distance of the *T. parva* isolates on Tp2 genome sequence and geographical locations of the farms was not observed (Figure 6).

### 3.5 Factors associated with *Theileria parva* positivity at the farm-level

The farm-level *T. parva* prevalence was not significantly different between the three districts studied (Table 4). However, the farm-level prevalence was marginally different among the nine EPAs studied in the three districts (*χ*^2^ = 15.438, df = 8, *p* = 0.051, Figure 3, the boundaries of EPAs are not shown). The farm-level *T. parva* positivity was significantly higher in the farms conducting extensive grazing (81.9%, 59/72) than semi-intensive farms (42.9%, 3/7, Fisher’s exact test, *p* = 0.035). On the other hand, there were no statistical associations between the farm-level *T. parva* positivity, and herd size, presence of ticks on cattle, or practice of tick control, respectively (Table 4). Among the farms that conducted tick control (*n* = 24), *T. parva* positivity was the highest in the least frequent tick-controlling group (90.0%) and the lowest in the most frequent group (40.0%). However, the farm-level *T. parva* positivity was not significantly different between tick-control frequency categories (Table 4).

**Table 4:** Results of univariable risk factor analysis for *Theileria parva* at the farm level.

Variable	Number of farms	*T. parva* infected farms	Prevalence (%)	*p*-value
District (*n* = 79)				
Chitipa	22	19	86.4	0.604
Karonga	13	10	76.9	
Mzimba	44	33	75.0	
Livestock system (*n* = 79)				
Extensive grazing	72	59	81.9	0.035
Semi-intensive	7	3	42.9	
Herd size (*n* = 79)				
<10	18	13	72.2	0.716
10–19	42	34	80.9	
≥20	19	15	78.9	
Presence of ticks on cattle (*n* = 79)				
Present	52	64	81.3	0.294
Absent	10	15	66.7	
Tick control (*n* = 79)				
Conducting	24	17	70.8	0.427
Not conducting	55	45	81.8	
Frequency of tick control (*n* = 24)				
Fortnightly	5	2	40.0	0.116
Monthly	9	7	77.7	
Once in 2 months	10	9	90.0	

Among the farms raising *T. parva* detected cattle, the mean within-herd *T. parva* prevalence was not significantly different between farms conducting extensive grazing (36.0%, *n* = 59 farms) and those conducting semi-intensive farming (37.6%, *n* = 3 farms, difference of logit = 0.155, SE = 0.439, *p* = 0.724). The mean within-herd *T. parva* positivity rate was not significantly different between farms that practiced tick control (25.6%, *n* = 17) and farms that did not (29.5%, *n* = 45, difference of logit = 0.018, SE = 0.228, *p* = 0.938).

### 3.6 Factors associated with *Theileria parva* positivity at the animal-level

There were no statistical differences in animal-level prevalence of *T. parva* across the three districts, sex, or cattle breeds (Table 5). The *T. parva* detection rate was significantly different between growth stages (Table 5). In multiple comparisons, calves (37.0%, 47/127) had significantly higher prevalence than adults (22.1%, 43/195, *p* = 0.016), but there were no significant differences between calves and heifers/young bulls (28.9%, 66/228, *p* = 0.265) or between heifers/young bulls and adults (*p* = 0.265). The mean BCS did not differ significantly between *T. parva*-positive (2.884) and -negative cattle (2.885, Wilcoxon rank-sum test, *W* = 29,701, *p* = 0.626).

**Table 5:** Results of univariable analysis of the factors associated with *Theileria parva* positivity at the animal level.

Variable	Number of cattle	Number of *T. parva* positive cattle	Prevalence (%)	*p-*value
District				
Chitipa	129	44	34.1	0.205
Karonga	92	27	29.3	
Mzimba	329	85	25.8	
Age				
Calves (≦5 m)	127	47	37.0	0.014
Weaners (6 m–1 y)	228	66	28.9	
Adults (>1 y)	195	43	22.1	
Sex				
Male	248	69	27.8	0.873
Female	302	87	28.8	
Breed				
Malawi zebu	506	146	28.9	0.156
Friesian pure	8	0	0	
Friesian cross	31	10	32.3	
Bosmara cross	5	0	0	
Treatment*				
Oxytetracycline	108	30	27.8	0.008
*Synadenium*	58	12	20.7	
Diseased but not treated	31	16	51.6	

*The data include only cattle that showed clinical signs suggestive of East Coast fever in the past.

Among animals on farms that reported the presence of signs indicative of ECF, the *T. parva* positivity rate was significantly different between three treatment groups: (1) ECF symptoms were reported but not treated, (2) infected cattle were treated with OTC, and (3) infected cattle were treated with *S. cupulare*. The multiple comparisons showed that the positivity rate was significantly higher among non-treated cattle (51.6%, 16/31) than cattle treated with OTC (27.7%, 30/108, *p* = 0.046) or *S. cupulare* (20.7%, 12/58, *p* = 0.018), but there was no difference between animals treated with OTC and those treated with *S. cupulare* (*p* = 0.415). The *T. parva* positive rate of animals that had not shown signs indicative of ECF (27.8%, 98/353 animals, not shown in Table 5) was similar to those of OTC and *S. cupulare* treated groups.

In the final multivariable generalized linear mixed-effects model, the remaining factors were age and types of treatment (Table 6). Regarding cattle age, the odds of *T. parva* positivity were significantly lower in adults (odds ratio: OR = 0.47, *p* = 0.003) compared with calves, but not for weaners (OR = 0.78, *p* = 0.316). Regarding treatment, the odds of *T. parva* positivity were significantly higher in untreated cattle with symptoms of ECF (OR = 2.38, *p* = 0.027) compared with asymptomatic cattle, but no significant increase was observed for those treated with oxytetracycline (OR = 0.99, *p* = 0.971) or *S. cupulare* (OR = 0.59, *p* = 0.133).

**Table 6:** Results of the final multivariable generalized linear mixed-effects model for the factors associated with *Theileria parva* positivity at the animal level.

Variable	Odds ratio	95% confidence interval	*p*-value
Age			
Calves (≦5 m)	Reference (1.00)	–	–
Weaners (6 m–1 y)	0.78	0.47–1.28	0.316
Adults (>1 y)	0.47	0.29–0.77	0.003
Treatment			
No symptom	Reference (1.00)	–	–
No treatment	2.38	1.11–5.12	0.027
Oxytetracycline	0.99	0.60–1.64	0.971
*Synadenium cupulare*	0.59	0.30–1.17	0.133

## 4 Discussion

This study clarified the molecular epidemiology and factors associated with *T. parva* positivity at the farm- and animal-levels in northern Malawi, and evaluated the ethnoveterinary efficacy of *S. cupulare* against ECF.

In the studied districts in the Northern Region of Malawi, *T. parva* was endemic (farm-level prevalence 78.5%), and the spatial distribution of *T. parva* infected farms was homogenous. Although Moran’s *I* in the Karonga District suggested a global clustering of *T. parva* negative farms, three *T. parva* negative farms located nearby each other along Lake Malawi had different farming styles and it was unlikely that the small area is free from ticks harboring *T. parva*. This high farm-level prevalence was comparable to 83.3% in southwestern Uganda in 2017 (Uchida et al., 2020). The distribution of ticks is predominantly determined by vegetation cover, climate, and abiotic factors such as temperature, humidity, and rainfall (Howell et al., 1978). In our study, sampling was conducted only in dry seasons to minimize bias of seasonal variation in tick abundance. However, further analysis on the associations between within-farm prevalence and ecological factors was not conducted as detailed ecological data were not collected. The animal-level prevalence of 28.4% in northern Malawi was similar to 30.1% in the indigenous breed cattle in the other highly endemic areas including various agro-ecological zones in Uganda between 2011 and 2012 (Kabi et al., 2014), and 45.0% in dairy cattle in southwestern Uganda in 2017 (Uchida et al., 2020). In contrast, a lower *T. parva* prevalence at the animal level 15.5% has been reported in southern Malawi, where incursion and slow expansion of *T. parva* into previously endemic areas is reported (Chikufenji et al., 2024). Low animal-level *T. parva* prevalence, 6.2%, has been reported in Tanzania as well, where hand spraying and acaricide dipping are commonly practiced, even though there are interactions between cattle herds and between cattle and wildlife (Haji et al., 2022).

The present study confirmed the circulation of genes encoding Tp1 and Tp2 in the cattle population in all three districts. Phylogenetic analyses demonstrated that Tp1 sequences formed a low-divergence, cattle associated clade, suggesting a close genetic relationship and the presence of conserved sequence motifs within the Tp1 gene among *T. parva* isolates from northern Malawi. This pattern is consistent with previously reported Tp1 variant clustering in cattle (Chatanga et al., 2020). Notably, sequences obtained in this study clustered with the reference sequence LC522068, which was described by Chatanga et al. (Chatanga et al., 2022) as an allele also identified in the Kasungu District in central Malawi, bordering Mzimba District to the south. This clustering suggests a regional distribution pattern and potential gene flow between these districts. The short branch lengths observed within the Tp1 clade indicate a minimal genetic distance, which may reflect recent evolutionary divergence, a shared epidemiological origin (e.g., movement of infected cattle or vaccination related strain circulation), or similar immune selection pressures acting within the same host species. In contrast, one Tp2 variant, Var-63 (sample “bovine Malawi PX418020” from Mpata EPA in Karonga District), appeared to harbor the most divergent isolate, as indicated by the multiple sequence alignment. This isolate may thus represent an outlier or regionally adapted strain. The presence of multiple amino acid differences across predicted epitope regions suggests potential implications for immune recognition and immune evasion. Tp2 sequences generated in the current study formed a clade with previously reported Tp2 variants PX418019 and PX418017 cluster together with moderate bootstrap support (62%), while PX418020 clusters with related East African isolates with strong support (100%), suggests reasonable confidence in this grouping and reflects a relatively high level of conservation among cattle derived Tp2 alleles. These variants are likely to be under host immune pressure, which may permit limited polymorphisms while maintaining a tight evolutionary cluster. No association between genetic distance in Tp2 and geographical locations of the farms suggested that a variety of *T. parva* variants may be homogenously co-exist in northern Malawi probably due to frequent animal movements including grazing, migration, and trading. In contrast, sequences derived from *Buffalo lawrencei Kenya* formed a distinct and highly divergent clade, consistent with previous findings demonstrating greater genetic diversity among buffalo-derived Tp2 alleles. The longer branch lengths observed in these sequences indicate substantial sequence divergence, highlighting the role of buffalo as reservoirs contributing to antigenic variability in *T. parva*. The Tp2 alleles from cattle in sample of the present study were found to be less diverse, possibly due to clonal propagation, host-imposed selective bottlenecks. By contrast, Tp2 alleles derived from buffaloes exhibited longer branches, indicative of broader diversification, an ancestral origin, and the potential emergence of novel antigenic variants. These findings have important implications for cross-species transmission dynamics. While cattle-specific Tp2 variants may represent suitable targets for CD8^+^ T-cell vaccines, insufficient coverage of buffalo-derived antigenic diversity could compromise vaccine efficacy in regions where cattle and buffalo co-graze. Based on the relatively small number of successfully sequenced samples (Tp1: *n* = 28, Tp2: *n* = 37), the full extent of genetic diversity of the Tp1 and Tp2 encoding genes in northern Malawi may not have been fully unrevealed, and the observed variants may not be entirely representative of circulating *T. parva* genotype. Future studies incorporating additional molecular markers, such as minisatellites, microsatellites, and genome-wide SNP analysis, would provide greater resolution for elucidating the populations and genetic diversity of *T. parva* in northern Malawi.

In our study, extensive farming and age of cattle were factors positively associated with *T. parva* positivity at the farm- and animal levels, respectively, and history of treatment both by OTC and *Synadenium* was a factor negatively associated. Grazing cattle are exposed to ticks harboring *T. parva*. Although cut and carry forage is sourced from natural pastures, the risk of carrying ticks to semi-intensive farms with forage may be limited (Maloo et al., 2001). The statistical non-significance in the within-herd prevalence between extensive grazing and semi-intensive farms suggested that once infected ticks are introduced to a farm, tick infestation can occur at a similar extent. A statistically significant difference in prevalence was recorded between young cattle and adults, suggesting that younger animals are more susceptible to *T. parva* than adults, findings consistent with those reported by Gitau et al. (1999) and Guilbride and Opwata (1963). High *T. parva* prevalence in calves suggested the importance of young animals as reservoirs of *T. parva*. Tick control was not associated with the farm-level *T. parva* positivity in this study. The information on the difference of tick control between growth stage of animals was not collected in this study, and future study should consider this. It may be strategic to prioritize acaricide spraying for calves. It should also be noted that most *T. parva* positive cattle sampled were described as healthy during the sampling period. Therefore, the apparently healthy but *T. parva*–positive animals might be recovered carriers with a low level of parasitemia. There are reports of higher *T. parva* detection rates in cattle populations raised under suboptimal vector control (Miyama et al., 2020) and vaccination against ECF (Radley et al., 1975). Again, future study should collect more detailed information on tick control practice. The cattle sampled in this study had never received an ECF vaccine, but vaccine trials were ever conducted in the region by Malawi government. More studies are needed for the acceptance rate and effectiveness of ECF vaccine, as well as distribution of *T. parva* genes originated from the vaccines circulating within cattle populations in the region.

In this study, 31.6% of farms used *S. cupulare*, a traditional mean of controlling ECF, which was at much higher rate than 3.6% reported by Kipronoh (2009) in Kenya. The present study is the first epidemiologic survey to report the use of ethnoveterinary treatment in the management of ECF in Malawi. Traditional veterinary practices have been applied for centuries to maintain optimal animal health, and these practices are often passed down from generation to generation (Ngeh et al., 2007). Tabuti et al. (2003) reported that *S. grantii* and *Asparagus racemosus* are used as remedies for ECF in Bulamogi, Uganda. This shows that knowledge of ethnoveterinary medicine (EVM) practices is widespread across Africa, including Malawi. There was no global spatial clustering in the use of *S. cupulare* which suggested that the plant is available and the treatment practice is common across the study areas. The final multi-variable generalized linear mixed-effects model results suggested that there was no association of the types of treatment (i.e., traditional ethnoveterinary using *S. cupulare* or modern chemical treatment using OTC) in reducing the animal-level prevalence to at the same level of the animals without suggestive symptoms of ECF. However, this epidemiological analysis employed a cross-sectional study, and could not determine the effect of *S. cupulare* by the longitudinal observation. To determine the effect, *in vitro* experiment and longitudinal epidemiological study are necessary. In Malawi, the latex and extracts of *S. cupulare* are utilized to treat parasitic infestations, inflammation, skin infections and gastrointestinal ailments in livestock. The pharmacological properties are primarily attributed to diterpenoids, triterpenes, and flavonoids, which exert antimicrobial, anti-inflammatory and antiparasitic effects (McGaw and Eloff, 2007). Just like any other drugs, application of *S. cupulare* during early stages of *T. parva* infection may be vital in practice. Phytochemicals make it suitable for topical applications and oral decoctions, though care must be taken due to potential toxicity (Katerere and Luseba, 2010). Therefore, sharing information of EVM, including traditional application practices, may be immediately helpful for farmers in Malawi, as well as in the other countries where suffer from ECF. Experimental researches on phytochemical properties of *S. cupulare* are needed for future drug development against *T. parva*.

There are limitations in this study other than mentioned earlier. The present study conducted interviews using a questionnaire. The responses to the questions regarding symptoms or treatment within 12 months might not be accurate due to recall bias. The selection of animals was purposive and it might introduce bias, although the sample age structure was controlled to represent the farms as much as possible.

EVM is increasingly recognized as a viable alternative or complementary system for managing livestock diseases in resource-limited settings. The strengths of EVM include affordability, accessibility, cultural relevance, and sustainability. However, concerns persist regarding standardization, efficacy validation, and dosage safety and more studies are required. The cross-tabulation analysis in the present study showed that the *T. parva* prevalence did not differ significantly between animals on farms that conducted grazing and those that conducted cut and carry feeding regarding different treatment options. This finding, together with those discussed above, might be indicative of farmers’ traditional knowledge, including knowledge regarding the use of traditional breed animals in overcoming the risks associated with ECF.

## 5 Conclusion

The Northern Region of Malawi was found to be highly endemic with spatial homogeneity in *T. parva* prevalence in cattle. However, molecular analysis suggested transmission patterns between cattle populations. *T. parva* was more prevalent in extensive farms and in calves, than semi-intensive farms and heifers and adults, respectively. Tick control programs should focus on the risky cattle populations. This study suggested that there is no association of the types of treatment, either traditional ethnoveterinary therapy using *S. cupulare* or modern chemical treatment using OTC in reducing the animal-level prevalence to the same level of the animals without suggestive symptoms of ECF, although *in vitro* experiment and longitudinal epidemiological study are necessary to determine the effect of *S. cupulare*. This study shed a light on enhancing ethnoveterinary practice against ECF, which may strengthen the sustainable and affordable disease control strategies, and reducing antimicrobial use.

## Data Availability

The data presented in the study are deposited in the NCBI GenBank repository, and the accession numbers are listed in the Supplementary table 1. The field data supporting the conclusion of this article will be made available by the authors, without undue reservation.
