# Supplementary material for: Molecular epidemiology of Theileria parva in cattle, factors associated with the positivity in northern Malawi, and efficacy of traditional medicine
Source: Front Microbiol. 2026 Aug 28;17:1826781. doi: 10.3389/fmicb.2026.1826781 (PMC13561776; doi:10.3389/fmicb.2026.1826781)
Supplement: Supplementary file 1 [file Table_1.DOCX]

**Supplementary table 1. Genetic data reported in this study**

| Repository | Accession number | URL |
| --- | --- | --- |
| GenBank (NCBI) | PX418011 | https://www.ncbi.nlm.nih.gov/nuccore/PX418011 |
| GenBank (NCBI) | PX418012 | https://www.ncbi.nlm.nih.gov/nuccore/PX418012 |
| GenBank (NCBI) | PX418013 | https://www.ncbi.nlm.nih.gov/nuccore/PX418013 |
| GenBank (NCBI) | PX418014 | https://www.ncbi.nlm.nih.gov/nuccore/PX418014 |
| GenBank (NCBI) | PX418015 | https://www.ncbi.nlm.nih.gov/nuccore/PX418015 |
| GenBank (NCBI) | PX418016 | https://www.ncbi.nlm.nih.gov/nuccore/PX418016 |
| GenBank (NCBI) | PX418017 | https://www.ncbi.nlm.nih.gov/nuccore/PX418017 |
| GenBank (NCBI) | PX418018 | https://www.ncbi.nlm.nih.gov/nuccore/PX418018 |
| GenBank (NCBI) | PX418019 | https://www.ncbi.nlm.nih.gov/nuccore/PX418019 |
| GenBank (NCBI) | PX418020 | https://www.ncbi.nlm.nih.gov/nuccore/PX418020 |
